# The Effect of Hypertension Duration and the Age of Onset on CV Risk Factors Expression in Perimenopausal Women

**DOI:** 10.1155/2019/9848125

**Published:** 2019-12-09

**Authors:** Ljiljana Trtica Majnarić, Ivo Martinović, Šefket Šabanović, Stjepan Rudan, František Babič, Thomas Wittlinger

**Affiliations:** ^1^Faculty of Medicine, University Josip Juraj Strossmayer, Department of Internal Medicine, Family Medicine and the History of Medicine, Osijek, Croatia; ^2^Faculty of Dental Medicine and Health, University Josip Juraj Strossmayer, Department of Public Health, Osijek, Croatia; ^3^Department of Cardiovascular Surgery, Philipps University of Marburg, Baldinger Straße 6, Marburg 35043, Germany; ^4^Department of Cybernetics and Artificial Intelligence, Faculty of Electrical Engineering and Informatics, Technical University of Košice, Letná 9, Košice 04200, Slovakia; ^5^Asklepios Harzkliniken, Department of Cardiology, Köslinerstr 12, Goslar 38642, Germany

## Abstract

**Background:**

The impact of hypertension duration and the time of onset on the expression of metabolic syndrome (MS) and other CV risk factors, in perimenopausal women, have not been studied so far. Methods. A total of 202 women, old 47–59 years, and diagnosed with hypertension, were recruited from primary care practices in eastern Croatia. The categories of hypertension duration were defined as <5, 5–10, and >10 years. Data were analyzed by standard statistical procedures.

**Results:**

The proportion of women with MS increases in parallel with hypertension duration (*p* = 0.025). Among the examined CV risk factors, significant increase in parallel with hypertension duration was found for body mass index (*p* = 0.007) and triglycerides (*p* = 0.07). The highest proportion of women with diabetes duration of less than 5 years, indicating recent diabetes onset, was found in the category of hypertension duration of less than 5 years, corresponding with the onset of hypertension in the time around menopause (*p* = 0.003). The strongest linear correlations with BMI and waist circumference were found for total serum cholesterol (*r* = 0.355 and 0.499, respectively).

**Conclusion:**

Hypertension onset at the time around menopause appears together with abdominal obesity and may be a driving force for CV risk factor accumulation in postmenopausal women.

## 1. Introduction

Hypertension, as the main cardiovascular (CV) risk factor, has its major impact on the development of CV disease and its common occurrence is in the older population [1, 2]. This effect of hypertension is the result of the synergistic action of a wide range of the concomitant CV risk factors and the influence of hypertension on the development of renal function decline, which, in turn, further amplifies progression of CV disease [3, 4].

There is a close association between hypertension and increased body weight and in their effect on the development of CV disease [5, 6]. Increased body weight can cause an increased blood pressure and reversely, individuals of equal weight, who had a higher blood pressure at the baseline, are likely to gain more weight in the future [7, 8]. Increased blood pressure shows even a stronger association with the central (abdominal) type of obesity than with general obesity [9]. It is best illustrated by the fact that hypertension is a major component of the metabolic syndrome (MS), a cluster of CV risk factors which include abdominal obesity, glucose intolerance or diabetes type 2, and dyslipidemia characterised by increased triglycerides and reduced HDL-cholesterol [10]. The development of CV disease could be amplified when MS is associated with hypertension [11].

There is a gender-related difference in the life course of hypertension, which is thought to influence the difference in terms of how men and women attain CV disease [12]. In men, incident hypertension starts to rise after adolescence and steadily increases with age [12]. In women, the critical period when hypertension starts to emerge is the transition from pre- to postmenopause, which occurs about the age of 50 [13]. Premenopausal women develop the peripheral type of obesity more frequently, characterised by subcutaneous fat accumulation, while men and postmenopausal women are more prone to the central type of obesity [12, 13]. The consequence of this switch in the type of obesity is that hypertension occurs more frequently in postmenopausal women as a part of MS than as an isolated disease [14].

The higher incidence of hypertension in postmenopausal women, than in premenopausal ones, raises the attention on the pathophysiological mechanisms that are involved in postmenopausal hypertension and that outweigh those involved in premenopausal hypertension [15]. The term menopause refers to the period of one year after the final menstrual bleeding. The term menopausal transition is used to assign a period around menopause, lasting for several years, when women experience intensive physiological and behavioural changes, which appear secondary to the decline in the reproductive hormones estrogens and are coincidental to chronological aging [16]. Due to the fact that estrogens have a vital role in energy metabolism and demonstrate vasodilatatory effects, which may protect premenopausal women from the development of hypertension and CV disease, a decrease in their circulating levels, in years after menopause, and disturbed estrogens to androgens ratio, lead to metabolic, inflammation, and vascular changes, which can contribute to changes in body composition and can trigger the rise in blood pressure and other CV risk factors [17–21]. The two critical periods during the menopausal transition when CV risk factors more intensively emerge include the late menopausal transition, a period that precedes the final menstrual bleeding by 3 to 4 years, and early postmenopause, a period until 5 years after menopause [16, 21].

Weight gain is a major health concern for women at midlife. Evidence from prospective cohort studies and animal models indicates that the hormonal changes during the menopausal transition are associated with abdominal fat deposition rather than general obesity [22]. Factors such as anxiety, depression, and sleep disorders, which can be due to both, hormonal and psychosocial reasons, have been reported to contribute to weight gain in menopausal women, including both, increased BMI and abdominal obesity associated with MS [23, 24].

If already present in the middle age, hypertension may cause significant reduction in life expectancy and more years living with CV disease [25]. Obesity and hypertension usually appear together in this age and are known to share common environmental risk factors, including bad lifestyles, chronic psychological stress, and unfavourable socioeconomic factors, which can serve as the basis for early CV prevention [26]. The effect of hypertension duration and the age of onset, on the accumulation of CV risk factors, among menopausal women, have not been studied so far. The object of this study is to determine how differences in hypertension duration and the age of onset, in hypertensive perimenopausal women, affect the expression of the components of MS and other major CV risk factors, for which evidence indicates their close association with hypertension, such as increased serum cholesterol [3].

## 2. Materials and Methods

This is a cross-sectional, proof-of-concept study. It was conducted in the primary care (PC) setting, during a-year period (2015), in an urban-rural area (small town) of eastern Croatia. Participants were recruited from six PC practices, all of which are located in the same health centre. The eligibility criteria included female gender, age 47–59 years, and with hypertension-diagnosed subject. The target age period was defined according to the evidence indicating 54 years as the median age of menopause of women in EU countries [27]. Taking into account that components of MS and other CV risk factors mostly emerge during late menopausal transition and the first three years of postmenopause, the eligible period for participants selection included 4 years prior to the last menstrual bleeding and 3 years after menopause or the age range of 49–57 years [16, 21]. We used a wider age range of 47–59 years (the average age was 52/53 years).

The primary sample, selected according to the defined criteria, included 224 women. Five of them, who reported surgically induced menopause, and another one, who used hormone replacement therapy, were excluded from the study. Fourteen women did not respond to the call for the recruitment. For two cases, data were incomplete. Thus, the final number of women, included in the study, was 202.

The Institutional Review Board of the Medical Faculty of the University of Osijek approved the study, and all participants gave their written informed consent.

Data for the study were used from PC electronic health records (eHRs). In Croatia, eHRs were established in PC services four decades ago and have been boosted several times, to achieve a high level of efficacy in managing patient care [28]. Some of these achievements include e-referrals to a PC laboratory, the list of diagnoses, prepared according to the ICD-10 code (International Statistical Classification of Diseases and Related Health Problems, 10^th^ Revision) and the chronic disease surveillance and preventive checkup systems. These systems require regular updates of laboratory data.

The selected women were invited by phone, or per short message service (SMS), and they were asked for anthropometric measurements when attending regular encounters.

The laboratory parameters, needed for this study, were only routinely collected data, used from PC laboratory templates or the panels for supporting chronic disease surveillance or preventive checkups. These parameters included fasting blood glucose (Fglu), triglycerides (TG), total serum cholesterol (Cho), LDL-cholesterol (LDL), HDL-cholesterol (HDL), and serum creatinine (Table 1).

Glomerular filtration rate (GFR), a measure of renal function decline, was derived from data indicating sex, age, weight, and serum creatinine and calculated according to the MDRD (the Modification of Diet in Renal Disease) formula, using the online calculation system [29]. Stages of chronic renal impairment were defined according to the US National Kidney Foundation Guidelines [30]. GFR < 90 > 60 mL/min/1.73 m^2^ was considered as mild renal impairment:

(1) $$\begin{array}{c} \text{MDRD formula GF }\left(\text{mL}/\min/1.73\;{\text{m}}^{2}\right)\\ =175\times \left(\text{Scr}\right)-1.154\times \left(\text{dob}\right)-0.203\times \left(\text{0.}742\text{ for female gender}\right). \end{array}$$

Blood lipid disorders were determined according to the ESC/EAS Guidelines (the European Society of Cardiology/the European Atherosclerosis Society) [31].

Anthropometric measures, height and weight, **for** BMI (kg/cm^2^)calculation, and waist circumference (wc), a measure of abdominal (central) obesity, were taken from participants during their encounters. Categories of BMI values were defined according to the WHO classification as BMI <19, malnutrition; BMI 19–24.9, normal weight; BMI 25–29.9, overweight; BMI ≥30, obesity [32].

In order to identify hypertensive women who also matched the criteria for the MS diagnosis, we used the NCP ATP III (the National Cholesterol Education Program Adult Treatment Panel III) definition. [33] This choice was made by medical doctors from our environment who are familiar with this definition which uses parameters that are available in PC eHRs.

NCP ATP III definition, an option for females:

1. Waist circumference ≥88 cm
2. Triglycerides ≥1.7 mmol/L
3. HDL-cholesterol <1.2 mmol/L
4. **Increased blood pressure (>130/85 mm·Hg)**
5. Fasting blood glucose > 6.1 mmol/L

Three out of five criteria indicate the MS diagnosis. As the diagnosis of hypertension was a part of the study selection criteria (bolded criteria of the NCP ATP III definition), two out of four other criterias of the NCP ATP III score indicated the MS diagnosis. A guarantee that hypertension is detected in its early stage was based on the fact that panels for preventive checkups also include screening for increased blood pressure. For the purpose of this study, hypertension duration was presented with categories, defined as <5, 5–10 and >10 years (*N* = 31,116,55). The average age of women in particular categories was as follows: 51.63 ± 2.18 SD, 52.23 ± 1.85 SD, and 53.64 ± 1.80 SD.

### 2.1. Statistical Analysis

Data were analysed using statistical software package IBM SPSS Statistics v24. Numerical data were presented as maximum and minimum values and averages and standard deviations (SD). Kruskal–Wallis's ANOVA test was used to assess differences in the examined CV risk factors. The level of significance of *p* < 0.05 was considered statistically significant.

Categorical data (proportions) were presented as relative frequencies and bar diagrams. Pearson's correlation coefficient was used to determine correlations between BMI and waist circumference and other CV risk factors.

## 3. Results

The values of parameters indicating components of MS and other CV risk factors that are usually concomitant to hypertension, including increased total serum cholesterol and LDL-cholesterol, decreased GFR (a measure of renal function), and increased BMI (a measure of body mass), are presented in Table 1.

Among the examined parameters, only BMI showed significant statistical changes (increase) in parallel with increased hypertension duration (*p*=0.007), while TG showed borderline statistical significance (*p*=0.07).

Linear correlations were assessed between BMI and waist circumference and all other examined CV risk factors. The best coefficients were achieved for the parameter total serum cholesterol (*r* = 0.355 and 0.499, respectively).

## 4. Discussion

**Figure 1:**
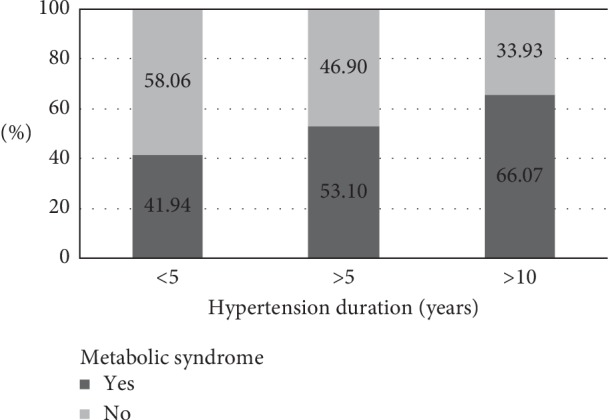
Proportions of women with and without metabolic syndrome according to categories of hypertension duration (<5 years, *N* = 31; 5–10 years, *N* = 116; >10 years, *N* = 55). It indicates that the proportion of women with metabolic syndrome, compared to those without, increases in parallel with increased hypertension duration. The differences are statistically significant (*p*=0.025).

The frequencies of MS in all three categories of hypertension duration, in this specific population group of hypertensive perimenopausal women, were found higher than were those reported in some other studies for perimenopausal women, even when only early postmenopausal (and not premenopausal) women were considered, as the early postmenopause has been recognized as the critical period for MS to emerge (Figure 1) [34, 35]. These frequencies are also higher than it has been reported in many studies for hypertensive patients and similar to high prevalence of MS that can be found in patients with uncontrolled hypertension or in senescent hypertensive patients, due to the fact that prevalence of MS increases with age [36, 37]. These frequencies are high and increase proportionally with increased hypertension duration (proportions of women with MS, relative to categories of hypertension duration, defined as <5 years, 5–10 years and >5 years, were 48%, 53.1% and 66.07%, respectively) (*p*=0.025) (Figure 1). An explanation for these results can be found in characteristics of the patient group that is highly vulnerable to MS, due to the synergistic interplay of hypertension, increased body weight, and menopause, all states where insulin resistance, a driving force of MS, is an underlying mechanism [13, 14, 38]. In this line of reasoning, there is also a result indicating a significant increase of BMI values along the categories of increased hypertension duration (Table 1, *p*=0.007). Following the increase in ratio of MS-related hypertension to isolated hypertension and a steady increase in BMI values, according to categories of increasing hypertension duration, there is also a significant increase in triglycerides values (*p*=0.07) (Table 1), another major component of MS.

Many women gain weight during the menopausal transition [22]. The women's reproductive function is closely associated with maintaining the body' energy homeostasis. The women' reproductive hormones, estrogens, thus play a vital role in the central regulation of the energy metabolism and body weight homeostasis, mainly by coupling the signals, in the hypothalamus, with the action of leptin, the main catabolic hormone in the brain. Due to the estrogen deficiency, postmenopausal women have increased risk for the development of leptin resistance and weight gain, even at normal leptin levels, such as the case in previously nonobese women [17].

Estrogens levels decline, due to the menopausal condition, is associated with the fat tissue redistribution, by favoring visceral (abdominal) over subcutaneous fat storage [17, 22]. Visceral adiposity is associated with increased inflammation, trigged by an array of inflammatory mediators that are released from visceral tissue in the circulation, including proinflammatory cytokines, such as IL-6 and TNF-alpha, chemotactic factors, and leukocytes adhesion molecules, which can subsequently lead to insulin resistance, impaired glucose tolerance, and possibly diabetes. [18, 39, 40] Apparently contradictory to this knowledge and to the above results, where the parameter BMI, a measure of general obesity was shown to significantly increase along with the increase in hypertension duration, the parameter waist circumference (wc), a measure of abdominal obesity and a cornerstone of MS, did not show this significant difference (Table 1). An explanation for this nonconcordant results lies in the fact that relatively higher (than expected) value of the wc measure characterises women with a hypertension duration <5 years, indicating recent hypertension onset, that is, in the time around menopause, compared to women with a longer hypertension duration. This disproportionate increase of wc, among women with the recent onset hypertension, is indicative of their high vulnerability for abdominal fat accumulation.

These results are in line with the concept of the common origin of vascular and metabolic disorders of MS, which have their background in the close association between insulin resistance and endothelial dysfunction [41]. In a short notice, visceral fat accumulation, due to menopausal decline in estrogens level, through its secretory activity, initiates both insulin resistance and endothelial dysfunction. Insulin resistance and secondary hyperinsulinaemia amplify endothelial dysfunction, mainly through the imbalance in the bioavailability of the vasodilator substances, such as nitric-oxide, and vasoconstrictor substances, such as endothelin-1. The endothelial dysfunction, by decreased flow in insulin-sensitive tissues, aggravates insulin resistance which, in turn, further advances the endothelial dysfunction. Increased insulin levels are thought to cause sodium retention, via kidneys, and increased sympathetic vascular tone. These disorders, operating in a vicious circle, subsequently lead to the development of both metabolic disorders known to characterise the MS and hypertension.

This view is also supported by the next result that indicates the lack of differences in glomerular filtration rates (GFRs), a measure of renal function decline, despite the increase in hypertension duration (Table 1). This nonconcordant result may be due to a smaller than expected GFR value in women with hypertension duration <5 years. This higher than expected negative effect of the recent onset hypertension on renal function can be explained by the simultaneous emergence of hypertension and increased body weight, during the period of menopausal transition, whereby both factors are known to affect the renal function [42]. In this term, evidence indicates that even in the absence of increased BMI or MS, the increase in androgens to estrogens ratio, in postmenopausal women, could play a role in the development of hypertension [20]. The proposed mechanism includes activation of the renin-angiotensin-aldosterone (RAS) system in the kidneys and other tissues, which may cause increase in blood pressure directly, by increasing the renal vascular resistance and body fluid volume and also by the stimulation of endothelin, oxidative stress, and inflammation.

If there is a preexisting increased body weight, these processes are amplified, making obese women to have a greater predisposition to hypertension-related target organ damage [11]. Decreased renal functional, from the earliest stages of chronic renal failure, can aggravate insulin resistance and hypertension [43].

**Figure 2:**
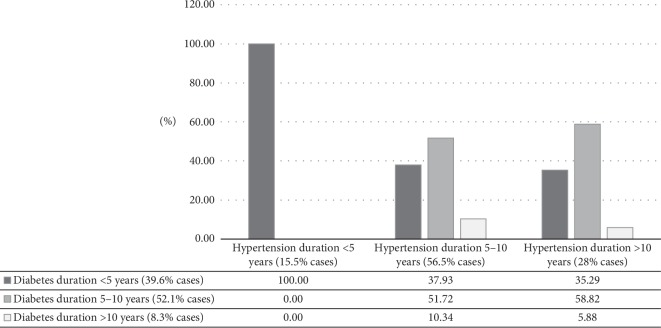
Categories of diabetes duration (<5, 5–10, >10 years) according to categories of hypertension duration (<5, 5–10, >10 years); patients diagnosed with diabetes mellitus type 2, *N* = 55. It indicates the relative frequencies (%) of categories of diabetes duration (<5, 5–10, >10 years) distributed across categories of hypertension duration (<5, 5–10, >10 years). The highest frequency (100%) was found for the category of diabetes duration of less than 5 years, indicating recent diabetes onset, in the category of hypertension duration of less than 5 years, compared to longer hypertension duration (Kruskal–Wallis ANOVA, *p*=0.003).

What is highlighted in this study is that hypertension onset at the time of menopausal transition, which appears together with increased body weight and abdominal fat accumulation, may be the main driving force in the CV risk factors promotion of subclinical organ damage [11, 12]. This assumption is further supported by results presented in Figure 2.

For the purpose of understanding the impact of hypertension duration and the time of onset on incident diabetes among perimenopausal women, we assessed the relationships between diabetes duration and hypertension duration.

By approximation of the time of diabetes onset, among women with a long-term (>10 years) diabetes duration (the estimated average age of this group of women is 53.6 years), it was possible to realise that diabetes rarely appears before the middle age (about 44 years of age). This is indicated by the small proportion (8.3%) of women in this category of diabetes duration (Figure 2). Even the presence of hypertension at this time of the women's life course cannot markedly influence diabetes development, as indicated by a very small proportion of women with a long-term diabetes duration (5.88%), in this subgroup of women with a long-term hypertension duration (Figure 2). While the new onset diabetes (<5 years of duration) can appear in all categories of hypertension duration, the highest rate of hypertensive patients with the new onset diabetes can be found in the category of women with the new onset hypertension (<5 years of duration) (Figure 2). And vice versa, these results suggest that a long-term existing diabetes (of 5–10 or >10 years of duration) is less likely to promote the onset of hypertension in women at the time of menopause, as indicated by the lack of these categories of diabetes duration among the group of women with the new onset hypertension (of <5 years of duration).

**Figure 3:**
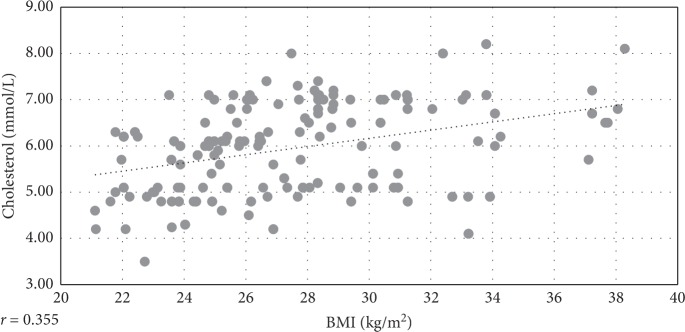
Linear correlation between BMI and total serum cholesterol in hypertensive menopausal women.

**Figure 4:**
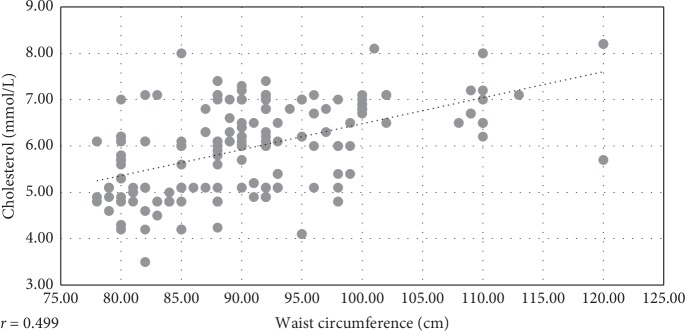
Linear correlation between waist circumference and total serum cholesterol in hypertensive menopausal women.

**Figure 5:**
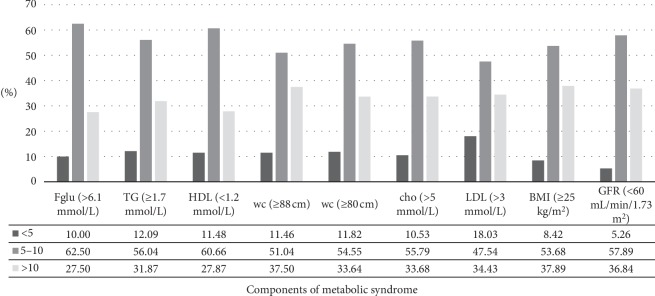
Relative frequencies of components of metabolic syndrome and other CV risk factors in hypertensive menopausal women according to categories of hypertension duration (<5, 5–10, >10 years). It shows the relative frequencies of particular components of MS and other CV risk factors among hypertensive menopausal women according to categories of hypertension duration (<5, 5–10, >10 years), presented graphically, as bar diagrams.

The analysis of correlations between metabolic CV risk factors and measures of body weight, BMI and waist circumference (wc), only showed clinically relevant correlations for total serum cholesterol (Figures 3 and 4). These results are unexpected according to the conventional definition of MS, where dyslipidemia associated with MS is characterised by increased TG and reduced HDL-cholesterol and not with increased total serum cholesterol, which indicate that there is a deviation of MS definition, to apply specifically to women at the age of menopause. This deviation is reflective of the fact that several factors, including decline in estrogens levels during the menopausal transition; hypertension and increased body weight, taken separately, may all increase serum cholesterol in perimenopausal women [44]. As evidence indicates, one of the first changes in CV risk factors, in perimenopausal women, include the rise in LDL- and total cholesterol [21]. That is likely to be also confirmed by the result showing a disproportionally higher rate of women with increased values of LDL-cholesterol (18.03%), in the category of women with the recent hypertension onset (<5 years), compared to rates of women with other increased CV risk factors, in this category of hypertension duration (Figure 5).

In this term, the discussion of this study could be considered as a part of the ongoing debate concerning whether MS is a cluster of cardiometabolic risk factors that is maintained stable among the adult population or it is a mixture of low related phenotypes that vary depending on characteristics of particular adult population groups, such as hypertensive perimenopausal women [13, 45, 46]. This dilemma arises from the fact that the available definitions of MS were created as the thresholds of increased CV risk in the general referent populations, and they may differently apply to different population groups, including both the composition of components of MS and their cutoff values [33].

The general implication of the results of this study is that both hypertension and diabetes, among women, most intensively emerge at the time around menopause. This conclusion is in line with the evidence indicating that hypertension among perimenopausal women usually appears as a part of MS [13, 35]. This further indicates the predisposition of perimenopausal women for the simultaneous appearance of MS and diabetes. Although evidence remains sparse in this respect, there are some indications that women with MS attain CV disease through the appearance of diabetes more often than men [47].

Taken together, cardiovascular and renal damage can be caused by multiple CV risk factors, emerging among women during menopausal transition, which indicate careful surveillance and early interventions. The both biological and behavioural changes, taking place during this transition, are responsible for these emerge in CV risk factors. Mood disorders, known to affect menopausal women, may lead to poor coping and unhealthy behaviours, and can accelerate the development of CV risk factors [22]. In this context, it would be possible to search for solutions on how to prepare effective interventions.

## 5. Conclusion

This small-scale study can be considered as a-proof-of-concept. The hypothesis emerging from this study, if further elaborated in future research, could have far-reaching consequences on planning CV prevention among females. It must be stressed, however, that CV risk factors, showing close associations with each other, are difficult to analyse in a cause-effect relationship, even in a longitudinal study design. As a possible research approach, the use of the time-dependent categories of major CV risk factors is suggested in this study. The lack of the measurement of reproductive hormones may attenuate the value of this study as the-proof-of-concept, because the interplay of the chronological age, the age of menopause, and the point of time within the menopausal transition, all dictate the emergency of CV risk factors in menopausal women. However, from the point of view of medical practice, this study clearly indicates that screening on the new onset hypertension, among women of the age range that covers the time of menopausal transition, the major part of the high risk women for developing CV disease, can be recognized and cured.

## Figures and Tables

**Table 1:** Values of parameters indicating components of metabolic syndrome and other CV risk factors in menopausal women according to categories of hypertension duration (<5, 5–10, >10 years) (*N* = 31, 116, 55).

CV risk factors	Min.	Max.	Average ± SD
*Hypertension duration <5 years*			
Fglu (mmol/L)	4.00	7.70	5.56 ± 0.79
TG (mmol/L)	1.05	4.49	1.64 ± 0.63
Cho (mmol/L)	4.20	8.00	5.75 ± 1.13
LDL (mmol/L)	1.05	5.00	2.98 ± 1.08
HDL (mmol/L)	0.80	2.42	1.32 ± 0.40
GFR (mL/min/1.73 m^2^)	50.38	99.46	72.08 ± 10.71
wc (cm)	78.00	110.00	89.13 ± 10.40
BMI (kg/m^2^)	21.77	37.22	26.55 ± 4.36
*Hypertension duration 5–10 years*			
Fglu (mmol/L)	4.50	8.80	5.76 ± 1.05
TG (mmol/L)	1.02	3.47	1.73 ± 0.39
Cho (mmol/L)	4.20	8.20	6.03 ± 0.87
LDL (mmol/L)	1.02	5.79	3.03 ± 1.06
HDL (mmol/L)	0.80	2.40	1.29 ± 0.36
GFR (mL/min/1.73 m^2^)	40.91	143.29	73.36 ± 15.65
wc (cm)	78.00	120.00	89.92 ± 8.49
BMI (kg/m^2^)	21.11	38.05	27.17 ± 3.83
*Hypertension duration >10 years*			
Fglu (mmol/L)	4.80	7.30	5.73 ± 0.61
TG (mmol/L)	0.42	4.27	1.90 ± 0.75
Cho (mmol/L)	3.50	8.10	5.90 ± 1.08
LDL (mmol/L)	2.00	5.70	3.32 ± 0.92
HDL (mmol/L)	0.80	2.60	1.42 ± 0.45
GFR (mL/min/1.73 m^2^)	47.54	95.68	71.25 ± 12.19
wc (cm)	80.00	120.00	92.30 ± 7.92
BMI (kg/m^2^)	22.72	38.28	28.89 ± 3.49

## Data Availability

The study data used to support the findings of this cross-sectional, proof-of-concept study are available from the corresponding author upon request.
